# Two cases of *Cupriavidus gilardii* infection presenting as pneumonia and cholecystitis were reported

**DOI:** 10.1097/MD.0000000000049476

**Published:** 2026-06-26

**Authors:** Han Qin, Bin Dong, Rui Qin

**Affiliations:** a Department of Sports Medicine, Heze Municipal Hospital, Heze, Shandong Province, China; b Department of Clinical Laboratory, Heze Municipal Hospital, Heze, Shandong, China.

**Keywords:** *Cupriavidus*, *Cupriavidus gilardii*, immunosuppression, opportunistic pathogen

## Abstract

**Rationale:**

*Cupriavidus gilardii* is an opportunistic pathogen that rarely causes clinical infections. Due to the absence of definitive antimicrobial susceptibility breakpoints established by the Clinical and Laboratory Standards Institute, diagnosis and treatment remain challenging. We report 2 cases of *C. gilardii* infection to enhance clinical recognition of this pathogen.

**Patient concerns:**

**Case 1:** A 72-year-old female with a history of hypertension, diabetes mellitus, and Parkinson disease presented with fever for > 10 days, accompanied by cough and chest tightness. Auscultation revealed coarse breath sounds bilaterally. **Case 2:** A 68-year-old female with a history of diabetes mellitus and sequelae of cerebral hemorrhage presented with fever for > 8 days, mild epigastric tenderness, and a positive Murphy sign.

**Diagnoses:**

Case 1 was diagnosed with pneumonia. Case 2 was diagnosed with cholelithiasis with acute cholecystitis.

**Interventions:**

Both patients received empirical antibiotic therapy. Sputum (Case 1) and bile (Case 2) cultures confirmed *C. gilardii* infection. Antimicrobial susceptibility testing guided targeted antibiotic adjustment in Case 1.

**Outcomes:**

Both patients became afebrile, showed clinical improvement, and were discharged.

**Lessons:**

*C. gilardii* infection should be suspected in immunocompromised or elderly patients with refractory fever, especially those with underlying neurological or cerebrovascular conditions. Early identification by MALDI-TOF MS and timely susceptibility testing are critical for guiding effective treatment, as this pathogen exhibits intrinsic resistance to multiple antibiotics, including carbapenems and aminoglycosides.

## 1. Introduction

Cupriavidus gilardii is a Gram-negative, oxidase-positive, non-spore-forming bacillus with peritrichous flagella,^[1]^ widely distributed in the environment.^[2]^ In recent years, this bacterium has garnered attention due to its ability to degrade herbicides^[3]^ and its resistance to heavy metals.^[4]^ Clinical infections caused by this organism have been sporadically reported. Herein, we report 2 cases of pulmonary infection and cholecystitis caused by *C. gilardii*, accompanied by a literature review.

## 2. Case reports

### 2.1. Case 1: Pneumonia caused by Cupriavidus gilardii

#### 2.1.1. *Patient presentation*:

A 72-year-old female presented to a local hospital with fever. Initial laboratory tests revealed a white blood cell (WBC) count of 14.68 × 10^9^/L and a C-reactive protein (CRP) level of 111.22 mg/L. After ineffective empirical treatment (details unspecified) at the local hospital, she was referred to our hospital. At admission, she reported a history of fever for over 10 days, accompanied by cough and productive sputum, as well as chest tightness. Her medical history included hypertension (managed with nifedipine), type 2 diabetes mellitus (managed with metformin), and Parkinson disease (managed with levodopa/benserazide). She had no history of smoking, alcohol use, or recent hospitalization.

#### 2.1.2. Diagnostic evaluation:

Auscultation revealed coarse breath sounds bilaterally without wheezes or crackles. Chest computed tomography revealed thickening and heterogeneous density of the bilateral bronchial walls, with a few ill-defined linear and patchy opacities in the lower lobes of both lungs, consistent with mild bilateral pulmonary inflammation. Laboratory tests showed WBC 13.84 × 10^9^/L. A comprehensive respiratory pathogen panel (adenovirus, coxsackievirus, Mycoplasma pneumoniae, Chlamydia pneumoniae, echovirus, influenza, parainfluenza virus types, respiratory syncytial virus, Legionella pneumophila) was negative. Differential diagnoses considered included:

Typical bacterial pneumonia – Less likely (no lobar consolidation); however, pathogens not on the panel cannot be excluded.

Viral pneumonia – Unlikely (negative for common viruses); other respiratory viruses not tested for remain possible.

Tuberculosis – Unlikely (no cavitation or apical disease); molecular testing not performed.

Fungal pneumonia – Unlikely (no immunocompromise, no nodular lesions); serologic and culture tests not available.

Aspiration pneumonia – Considered (Parkinson disease as risk factor); absence of dependent opacities and rapid treatment response argue against typical aspiration, but microaspiration could not be definitively excluded.

The workup was constrained by the lack of advanced microbiological testing (e.g., fungal studies, tuberculosis molecular assays, or bronchoalveolar lavage). Therefore, while several common etiologies were rendered unlikely, definitive exclusion of all alternative diagnoses was not possible. The final diagnosis was established based on the combination of clinical presentation, imaging, response to therapy, and subsequent identification of the causative pathogen (Cupriavidus gilardii) from sputum culture.

#### 2.1.3. Further examinations:

Sputum culture was performed on blood agar and MacConkey agar. After 48 hours of incubation at 35°C in ambient air, small, moist, gray-white colonies appeared. Matrix-assisted laser desorption/ionization time-of-flight mass spectrometry (MALDI-TOF MS, VITEK MS, bioMérieux) identified the isolate as Cupriavidus gilardii with 99.9% confidence (June 12). Antimicrobial susceptibility testing was performed using the broth microdilution method according to Clinical and Laboratory Standards Institute guidelines. The isolate was sensitive to cefoperazone-sulbactam, levofloxacin, and trimethoprim-sulfamethoxazole; intermediate to piperacillin-tazobactam, ceftriaxone, and minocycline; and resistant to cefazolin, ceftazidime, cefotaxime, aztreonam, imipenem, meropenem, gentamicin, amikacin, tobramycin, and ciprofloxacin.

#### 2.1.4. Treatment process and outcomes:

Empirical therapy with intravenous piperacillin-tazobactam was initiated based on clinical experience on June 6. The patient’s Parkinson disease and diabetes placed her at risk for aspiration and healthcare-associated pathogens, supporting broader coverage.

**Clinical and laboratory response:**

**June 6 (admission):** WBC 14.68 × 10^9^/L, CRP111.22mg/L

**June 9 (day 3 of piperacillin-tazobactam):** WBC 13.84 × 10^9^/L

**June 14 (discharge):** Patient was completely asymptomatic.

After culture results were available on June 12, antibiotic therapy was switched to intravenous levofloxacin based on susceptibility results.By June 14, the patient was afebrile without cough or chest tightness, and her condition improved. She was discharged.

Informed consent was obtained from the patient for publication of this case report.

### 2.2. Case 2: Cholecystitis caused by Cupriavidus gilardii

#### 2.2.1. Patient presentation:

A 68-year-old female presented to a local hospital with fever and right upper quadrant abdominal discomfort. Abdominal ultrasound revealed gallbladder enlargement with multiple stones. She received intravenous anti-inflammatory therapy (details unspecified) with slight symptomatic improvement and was transferred to our hospital for further management. At admission, she reported fever for over 8 days. Her medical history included type 2 diabetes mellitus and sequelae of cerebral hemorrhage. She denied alcohol use or prior biliary interventions.

#### 2.2.2. Diagnostic evaluation:

On admission (June 3), the patient was febrile. Abdominal examination showed a flat, soft abdomen with mild epigastric tenderness, no rebound tenderness or abdominal muscle rigidity, and a positive Murphy sign. Ultrasound upon admission revealed an enlarged gallbladder with multiple stones. No hepatosplenomegaly was noted.

#### 2.2.3. Further examinations:

Empirical therapy with intravenous ceftazidime was initiated on June 3. Ceftazidime was chosen for suspected biliary sepsis based on its excellent biliary penetration and coverage of Enterobacteriaceae and Pseudomonas species.

On June 4, ultrasound-guided percutaneous transhepatic gallbladder drainage (PTGBD) was performed, yielding turbid, purulent bile, which was sent for bacterial culture and Gram staining. On June 5, the aerobic blood culture bottle signaled growth after 18 hours. Gram staining of the bile and blood culture revealed Gram-negative bacilli. Subculture on Columbia blood agar yielded moist, gray-white colonies after 24 hours. MALDI-TOF MS identified the isolate as *C. gilardii* with 99.9% confidence. Unfortunately, antimicrobial susceptibility testing could not be performed due to the unavailability of the appropriate testing card in our laboratory at that time.

#### 2.2.4. Treatment process and outcomes:

Following PTGBD, the patient’s fever gradually subsided.

**Clinical and laboratory response:**

**June 4** WBC: 7.4 × 10^9^/L NEUT: 6.74 × 10^9^/L (within normal range but at the higher end)

**June 8 (day 5 of ceftazidime + PTGBD**) The patient was afebrile (temperature 36.4°C), without abdominal tenderness or a positive Murphy sign.

WBC decreased to 6.95 × 10^9^/L

NEUT decreased to 5.73 × 10^9^/L, demonstrating a favorable downward trend consistent with clinical improvement.

Note: CRP and PCT data were not available during the hospitalization for technical reasons.

After *C. gilardii* was identified, we specifically considered whether to change antibiotics. Because the patient showed clinical improvement (afebrile by day 5, decreasing WBC) and remained stable, therapy was continued without change. Given this favorable response and the patient’s stable condition, antibiotic therapy was continued without alteration. She was discharged on June 11 after her blood glucose levels stabilized (achieved on an insulin sliding scale) and she remained afebrile for 72 hours.

Informed consent was obtained from the patient for publication of this case report.

## 3. Discussion

*Cupriavidus gilardii* belongs to the genus *Cupriavidus* within the family *Burkholderiaceae.* It was first isolated and named *Ralstonia gilardii* sp. nov. by Coenye et al in 1999.^[5]^ In 2004, Vaneechoutte et al^[1]^ divided the genus *Ralstonia* into 2 lineages based on 16S rDNA sequence differences: the *Ralstonia eutropha* lineage and the *Ralstonia pickettii* lineage. They proposed reclassifying the *R. eutropha* lineage as *Wautersia* gen. nov. and described *Wautersia gilardii.* Later that year, Vandamme and Coenye,^[6]^ through DNA-DNA hybridization, phenotypic characterization, DNA base ratio analysis, and 16S rRNA gene sequencing, demonstrated that the genus *Wautersia* was synonymous with the genus *Cupriavidus*, named in 1987.^[7]^ Consequently, *Wautersia* species were reclassified under the genus *Cupriavidus*.

In recent years, *C. gilardii* has been isolated from various human tissues and is considered an opportunistic pathogen.^[8]^ Clinical cases of infection caused by this organism are summarized in Table 1.^[9–14]^

**Table 1:** Reported cases of *Cupriavidus gilardii* infection.

Year (ref) author	Patient	Underlying condition(s)	Symptom(s)	Source of isolation	Susceptibility (S/I/R)	Treatment	Outcome
This study	72, F	HTN, DM, PD	Fever	Sputum	**S:** SCF, LEV, SXT **I:** TZP, CRO, MH **R:** KZ, CAZ, CTX, AZT, IPM, MEM, AK, CN, TOB, CIP	TZP -> LEV	Improved
	68, F	DM, Stroke	Fever	Bile	ND	CAZ	Improved
2025 Liu et al.	87, M	Heart failure, Myocardial infarction	Fever	Sputum	**S:** SCF, FEP, TZP, CIP, LEV, DOX, MH, TGC, SXT **I:** CAZ **R:** IPM, MEM, AK, TOB	SCF + MH	Death
2024 Zhao et al.	81, M	-	-	BALF	ND	CAZ, AK, LEV, MXF, MEM, LZD	Death
	50, M	Immunosuppressant use	-	Ascitic fluid	ND	MEM, SCF, LEV	Improved
	73, F	Immunosuppressant use	-	Pleural fluid	ND	IPM, VA, SCF, TGC, MEM, TEC, LEV	Death
	68, M	-	-	Sputum	ND	VA, SCF, TGC	Improved
2023 Fang et al.	78, M	COVID-19, Glucocorticoid use	Dyspnea	BALF	ND	TZP -> IM/CI + SXT	Death
2017 Zhang et al.	87, M	COPD, HTN	Chills	Blood	**S:** SCF, FEP, TZP, CTX, CRO, IPM, CIP, LEV, MH, TGC, SXT, PRL **R:** MEM, AK, AMP, RIA	PRL	Improved
2016 Kobayashi et al.	90, F	Pacemaker implanted	Fever	Wound, Blood, Lead	**S:** FEP, MH, SXT **R:** CAZ, AZT, MEM, CN	CIP + SAM -> FEP -> MH	Improved
2014 Tena et al.	36, M	Immunosuppressant use (Renal transplant)	Thigh pain	Thigh abscess	**S:** CIP, LEV, CTX, TGC, SXT, CXM **R:** P, AMC, IPM, MEM, CN, TOB	CIP, MEM, DA	Improved
2010 Karafin et al.	12, F	Aplastic anemia	Fever	Throat, Stool, Blood	**S:** SXT, MXF, TE **R:** AK, AZT, CN, MEM, TOB, KZ, AMP, FOX, ETP	AK, LZD, MEM, FEP, CIP	Death
2001 Wauters et al.	7, F	Acute lymphoblastic leukemia	Fever	Blood	**S:** CAZ, AK, CRO, CXM, OFX **R:** AMP, TOB, CN, PRL, AZT	AMP + MET	Improved

AK = amikacin, ALL = acute lymphoblastic leukemia, AMC = amoxicillin-clavulanate, AMP = ampicillin, AZT = aztreonam, BALF = bronchoalveolar lavage fluid, CAZ = ceftazidime, CIP = ciprofloxacin, CN = gentamicin, COPD = chronic obstructive pulmonary disease, CRO = ceftriaxone, CTX = cefotaxime, CXM = cefuroxime, DA = clindamycin, DM = diabetes mellitus, DOX = doxycycline, ETP = ertapenem, FEP = cefepime, FOX = cefoxitin, HTN = hypertension, IM/CI = imipenem-cilastatin, IPM = imipenem, KZ = cefazolin, LEV = levofloxacin, LZD = linezolid, MEM = meropenem, MET = netilmicin, MH = minocycline, MXF = moxifloxacin, ND = not determined, OFX = ofloxacin, P = penicillin, PD = Parkinson disease, PRL = piperacillin, RIA = rifampin, SAM = ampicillin-sulbactam, SCF = cefoperazone-sulbactam, SXT = trimethoprim-sulfamethoxazole, TE = tetracycline, TEC = teicoplanin, TGC = tigecycline, TOB = tobramycin, TZP = piperacillin-tazobactam, VA = vancomycin.

### 3.1. Analysis of underlying conditions as risk factors

The 2 cases presented here highlight distinct predisposing mechanisms for *C. gilardii* infection. In **Case 1**, the patient’s Parkinson disease likely contributed to infection risk through multiple pathways. First, Parkinson disease is associated with dysphagia and impaired swallowing function, which increases the risk of silent aspiration – a well-established mechanism for pneumonia in this population.^[15]^ Second, impaired mucociliary clearance due to reduced cough efficiency may facilitate bacterial colonization of the lower respiratory tract.^[16]^ Third, the patient’s advanced age and comorbid diabetes mellitus further compromised her immune defense. These factors collectively may have created an opportunity for *C. gilardii*, an environmental organism, to establish pulmonary infection.

In **Case 2**, the patient’s sequelae of cerebral hemorrhage created different but equally important vulnerabilities. Immobility and reduced functional status lead to an imbalance in the immune system, resulting in immune dysfunction and increased infection susceptibility.^[17]^ It is notable that both patients had diabetes mellitus, a well-recognized risk factor for infections due to impaired neutrophil function and altered host immunity.^[18]^ The combination of neurological impairment (which may affect swallowing, mobility, or self-care) and metabolic dysfunction (diabetes) may synergistically increase susceptibility to opportunistic pathogens like *C. gilardii*. Clinicians should therefore maintain a low threshold for considering atypical pathogens in such patients presenting with refractory fever.

### 3.2. Antimicrobial resistance patterns

As shown in Table 1, infections caused by *C. gilardii* predominantly occur in immunocompromised or elderly individuals, with no significant gender predilection, and fever is a common presenting symptom. Isolates frequently demonstrate resistance to meropenem, tobramycin, and gentamicin. Ruiz et al^[19]^ suggested that *C. gilardii* possesses a homolog of the Pseudomonas aeruginosa MexAB-OprM efflux pump, which may contribute to resistance to meropenem and aminoglycosides. A novel aminoglycoside 3-N-acetyltransferase [AAC(3)-IVb, AacC10] may also reduce susceptibility to gentamicin and tobramycin. Most isolates are resistant to ampicillin, amoxicillin, and aztreonam, potentially due to a novel β-lactamase (OXA-837) that decreases susceptibility to ampicillin.^[19]^ Isolates are generally susceptible to trimethoprim-sulfamethoxazole, quinolones, minocycline, and cefepime, while resistance to amikacin, imipenem, and ceftazidime is variable. Misidentification by conventional diagnostic systems^[10]^ or failure to identify the species,^[11]^ coupled with its intrinsic resistance genes, pose significant challenges for clinical management. Notably, some strains can develop resistance to levofloxacin during treatment through gyrA gene mutations,^[8]^ underscoring the need for close monitoring of antimicrobial susceptibility during therapy.

### 3.3. Colistin resistance and public health implications

Studies indicate that *C. gilardii* may be a source of the *mcr-5* gene.^[20,21]^ Massip et al^[20]^ conducted in vitro drug sensitivity experiments on Cupriavidus species, which showed that only 67% (4/6) of *C. gilardii* were sensitive to colistin. Additionally, the ArnT enzyme (MCR phosphoethanolamine transferase) encoded by the *mcr-5* gene, which mediates colistin resistance, was detected in other Cupriavidus species. This gene has been frequently isolated from hospital water and sewage systems, suggesting aquatic environments serve as important reservoirs for the horizontal transfer of this gene among different bacterial genera and species.^[22]^ Therefore, accurate identification and effective treatment of this pathogen are not only clinically significant but also crucial for public health surveillance and control.

## 4. Conclusions

We report 2 cases of *C. gilardii* infection presenting as pneumonia and cholecystitis in elderly patients with underlying neurological disorders and diabetes mellitus. The diagnosis was confirmed by MALDI-TOF MS in both cases. Case 1 was successfully treated with levofloxacin guided by susceptibility testing, while Case 2 resolved with ceftazidime combined with source control (PTGBD). These cases highlight the importance of considering *C. gilardii* as a potential pathogen in immunocompromised or neurologically impaired patients with refractory fever. Early microbiological diagnosis and antimicrobial susceptibility testing are essential for optimal management.

## Author contributions

**Conceptualization:** Rui Qin.

**Project administration:** Rui Qin.

**Resources:** Rui Qin.

**Writing – original draft:** Han Qin.

**Writing – review & editing:** Bin Dong.
